# Review of "System approach to engineering design" by P.H. Sydenham

**DOI:** 10.1186/1475-925X-3-45

**Published:** 2004-12-03

**Authors:** Adrian Mondry

**Affiliations:** 1 Medical and Clinical Informatics Group, Bioinformatics Institute, Singapore

Based on a semester-long seminar on the topic, this book aims to fill a gap in the current engineering curricula by taking a wide-angle view at the process of engineering design rather than focusing on a more narrow and in-depth approach. As part of Artech House Publishers' technology management and professional development library, this is an excellent introduction to the topic and may serve for later reference.

The author, Peter. H. Sydenham is the inaugural professor and head of the University of South Australia's School of Electronic Engineering, co-founder of the Australian Centre for Test and Evaluation, and a director of Global Systems Engineering Consulting Pty Ltd. This brief biographical sketch explains his qualification to write a book of such practical value and dimension, and why the book is pedagogically sound.

The book, according to the preface, aims at those readers who are engineers "who have, or aspire to team leadership or want to take on increased team interfacing responsibilities". As such it builds on and expands what the author perceives to be too little taught in the regular university courses, and covers what is lacking: "breadth of the knowledge now needed to be an effective engineering designer."

Twelve chapters offer a step-by-step introduction to the topic. The first chapter, "Systems Thinking and Systems Engineering", gives an overview and the "philosophical" background, while the last looks at "Change and Future Trends". The remaining ten chapters cover the practical aspects of the whole process of systems engineering design with a more hands-on approach, including basics of supporting knowledge such as staff management from planning over recruitment through training to promotion or termination of contract (chapter 3) or information technology support. As such, chapters 2–4 cover the more general aspects, while chapters 5–9 cover the design process from idea to evaluation. Chapter 10 intersperses legal issues, while chapter 11 covers the prototype build, i.e. the step from abstract design to material product.

This is an introductory textbook, and as such, it does a very good job of the task at hand. The language is clear, the case examples are well chosen, and the message is conveyed without fail. Some issues are discussed somewhat at length; for example, the basic IT issues are explained in a very detailed way, but one might assume that the basics would be self-evident for today's user: the target audience will have grown up in the age of the personal computer and the internet and as such, will neither question the computer's usefulness nor be overly naïve with regards to the many IT bugs one has to deal with.

The illustrations are about as clear as can be-some, in fact, are not clear at all but as they are meant to demonstrate the complexity of the issue at hand, the message gets conveyed the way it should be.

How does this general book relate to the specialist field of biomedical engineering? To quote from p. 12: "Researchers in the life sciences were driven by a need to better understand how nature works and controls itself. Out of this pioneering work emerged general systems theory cybernetics, self-organizing systems, automation, automaton systems, organizational science, operations research, systems science, and more-topics with which engineers are not usually that familiar". This lack of familiarity becomes very evident when young engineers join research teams with a focus on applications for the life sciences, such as biomechanical engineering, but also within the broad field of bioinformatics, and they usually take more time to acquire and apply that holistic view of their work than life scientists need to acquire and implement detailed and circumscript engineering knowledge to accomplish their tasks. As such, this book can be recommended to engineers working in biomedicine even at the outset of their careers as it may draw their attention to the importance of this view of things in the new field they enter. Competing recent titles include works by Blanchard [1], Hitchins [2] and Sheridan [3]. A more general outlook on the importance of systems thinking in many areas of life, and far beyond engineering design, is Gharajedaghi's [4] modern classic.

One point that a next edition seriously needs to address is the text editing. Using a word processor does not guarantee a perfect text: this one has a lot of extra words, and as many words missing. As faulty as the text editing is the punctuation check. These errors occur as often as once per page, and force the reader to repeat the study of entire paragraphs several times because, more often than not, it is the missing preposition or punctuation that poses a serious threat to understanding. The publishing house would do well in employing an old-fashioned human text editor to spare the reader such nuisance.

In summary, this is a basic textbook to project design management for aspiring team leaders, not only in engineering but for any scientific and some business projects as well. Well written, this is a recommended introduction to the matter and may serve as a reference book and reminder even to the experienced team leader.

*Adrian Mondry is at Bioinformatics Institute, Singapore *Mondry@hotmail.com.
